# STK11 (LKB1) mutation suppresses ferroptosis in lung adenocarcinoma by facilitating monounsaturated fatty acid synthesis

**DOI:** 10.1515/med-2023-0845

**Published:** 2024-01-06

**Authors:** Qiang Zou, Bo Tang, Xianchao Chen, Chuang Zhang, Yun Huang

**Affiliations:** Department of Cardio-Thoracic Surgery, Zigong Fourth People’s Hospital, Zigong, Sichuan, 643000, China

**Keywords:** STK11, lung adenocarcinoma, monounsaturated fatty acid synthesis, ferroptosis

## Abstract

Serine/threonine kinase 11 (STK11), a tumor suppressor gene, exhibits frequent mutations in lung adenocarcinoma (LUAD). However, the specific molecular mechanisms by which STK11 mutations exert an influence on the biosynthesis of monounsaturated fatty acids (MUFAs) and subsequently affect ferroptosis in LUAD remain indistinct. In this study, bioinformatic analysis was employed to probe into the linkage between STK11 and key inhibitory genes of ferroptosis, namely SLC7A11 and SCD1, in LUAD tissues. Quantitative reverse transcription polymerase chain reaction was employed to assess the expression of STK11 in both wild-type and mutant STK11 LUAD cells, cell counting kit-8 to assess cell viability, and flow cytometry to detect apoptosis. A transmission electron microscope was utilized to observe mitochondrial morphology, and Western blot to ascertain the protein expression of STK11, ferroptosis-related proteins, and the enzyme SCD1 involved in MUFA synthesis. Oil red O staining was employed to test the distribution of lipid droplets in cancer cells, and a lipid quantification method to measure the content of MUFAs. Commercial kits were employed to assess the levels of lipid reactive oxygen species, malondialdehyde, glutathione, and Fe^2+^ in cells. The result revealed a negative correlation between STK11 and SLC7A11 as well as SCD1, with STK11 expression downregulated in mutant STK11 LUAD cells. Furthermore, STK11 mutations were found to suppress ferroptosis in LUAD cells by affecting MUFA synthesis. Subsequent rescue assays demonstrated that STK11 mutations hindered ferroptosis by impacting the synthesis of MUFAs in LUAD cells. This study provided evidence that STK11 mutations suppressed ferroptosis in LUAD cells by promoting MUFA synthesis, thus offering a novel research direction in the management of LUAD.

## 1 Introduction

Lung cancer (LC) is one of the most prevalent cancers globally, resulting in 2.2 million fresh incidences and 1.8 million fatalities in 2020, and emerging as a prominent contributor to cancer-associated mortality on a global scale [1]. Despite significant advancements in early screening techniques and treatment interventions, lung adenocarcinoma (LUAD) continues to be the predominant histological subtype of non-small cell LC, and its 5 year survival rate remains disheartening [2,3]. Hence, a pressing imperative exists to augment our comprehension of the molecular mechanisms that drive LC pathogenesis and to foster the development of novel therapeutic strategies.

Recently, a newly discovered regulated cell death pathway, known as ferroptosis, has been identified as a result of oxidative damage. Ferroptosis is featured by iron accumulation, lipid peroxidation, and subsequent plasma membrane rupture [4]. The involvement of ferroptosis in the progression and treatment response of various malignancies is increasingly supported by emerging evidence, offering significant potential in cancer treatment strategies [5]. For instance, in gastric cancer, STAT3 has been identified as a critical negative regulatory factor of gastric cancer ferroptosis, and its inhibition can suppress gastric tumor growth and alleviate 5-Fu chemotherapy resistance [6]. In glioblastoma, oxidized iron nanoparticles loaded with paclitaxel inhibit glioblastoma growth through an enhanced autophagy-dependent ferroptosis pathway [7]. In recent studies, miR-6077 has been found to promote cisplatin/pemetrexed resistance in LUAD cells by inducing cell cycle arrest through the CDKN1A-CDK1 pathway and protecting cells from cisplatin/pemetrexed-induced cell death through the KEAP1-NRF2-SLC7A11/NQO1-mediated ferroptosis pathway, both *in vitro* and *in vivo* [8]. As studies collectively indicated, promoting ferroptosis in cancer cells holds promise for tumor therapy. However, the regulatory mechanisms governing ferroptosis in LUAD cells remain incompletely understood and warrant further investigation.

Recent studies have shown a close linkage between fatty acid (FA) metabolism and ferroptosis [9]. Peroxidation of membrane lipids has been revealed as a central event in ferroptosis [10]. Polyunsaturated FAs are activated and incorporated into membrane phospholipids, inducing iron-dependent lipid peroxidation and driving ferroptosis [11]. However, monounsaturated FAs (MUFAs) are considered to protect cell membranes from the threat of ferroptosis [12]. Magtanong et al. [13] found that exogenous MUFAs effectively inhibit ferroptosis in mouse embryonic fibroblasts. However, there have been no reports on the impact of MUFA synthesis on ferroptosis in LUAD cells.

In LUAD, serine/threonine kinase 11 (STK11), also known as liver kinase B1 (LKB1), ranks as the third most commonly mutated gene after TP53 and KRAS and has been found in up to 33% of primary LUAD cases [14]. Numerous studies have demonstrated the linkage of STK11/LKB1 mutations with primary resistance to immune therapy in LC [15,16,17]. Additionally, concurrent mutations in STK11 and KEAP1 have been shown to foster ferroptosis protection and SCD1 dependency in LC [18]. However, whether STK11 mutation affects MUFA synthesis remains unexplored. The primary objective of this work was to delve deeper into the impact of STK11 mutation on the modulation of ferroptosis in LUAD through *in vitro* experiments, providing new directions for the treatment of LUAD.

Here, we elucidated the molecular mechanisms by which STK11 mutation suppressed ferroptosis in LUAD. We found that STK11 mutations in LUAD cells promoted the expression of the ferroptosis-inhibiting protein SLC7A11. Subsequently, STK11 knockout cells were generated, and cell functional experiments revealed that STK11 mutations suppressed ferroptosis in LUAD cells. Further rescue assays demonstrated that STK11 mutation mediated MUFA synthesis, thereby inhibiting ferroptosis in LUAD cells. This study provided insights for future researchers and offers new potential therapeutic targets in treating LUAD.

## 2 Materials and methods

### 2.1 Bioinformatics

mRNA data of LUAD samples, encompassing both tumor and non-tumor tissues, were supplied with The Cancer Genome Atlas database. The correlation between STK11 expression and key inhibitory genes of ferroptosis and MUFA synthesis enzymes was analyzed.

### 2.2 Cell culture

STK11 wild-type LUAD cell lines (Calu-3, H1299) and STK11-mutant LUAD cell lines (A549, H23, H2030) were got from American Type Culture Collection (ATCC, USA). H1299, H23, and H2030 cells were kept in RPMI-1640 medium with 10% fetal bovine serum (FBS), Calu-3 cells in EMEM medium with 10% FBS, and A549 cells in F-12K medium with 10% FBS. All cells were cultured in an incubator at 37℃ with 5% CO_2_.

### 2.3 STK11 knockout and overexpression

For STK11 knockout, GuideScan (www.guidescan.com), an optimized CRISPR design tool, was used to select single-guide RNAs (sgRNAs) targeting human STK11. sgRNAs with high target scores and low off-target effects were chosen. Oligonucleotides targeting the desired site were annealed and cloned into a plasmid using enzymatic digestion. The plasmids containing the target sgRNA sequences were transfected into Calu-3 and H1299 cells utilizing Lipofectamine 2000 (Invitrogen, USA).

For STK11 overexpression, oe-STK11 and the corresponding negative control (Ribobio, China) were transfected into A549, H23, and H2030 cells utilizing Lipofectamine 2000 Transfection Reagent (Invitrogen, USA).

### 2.4 Electron microscopy analysis

To observe the morphological changes in mitochondria after induction of ferroptosis, cells were put on four-chambered glass slides (Thermo Scientific, USA) at a density of 1.5 × 10^4^ cells per well and treated with or without Erastin for 24 h. The samples were fixed using a double fixation method (glutaraldehyde solution and 1% osmium tetroxide solution), followed by dehydration with a series of graded ethanol solutions (30, 50, 70, 80%). Subsequently, the samples were infiltrated, embedded, sectioned, and stained, and images were captured utilizing an Olympus EM208S transmission electron microscope (TEM).

### 2.5 Quantitative reverse transcription polymerase chain reaction (qRT-PCR)

Total RNA from cells was stemmed using TRIzol (Invitrogen, USA), and the RNA was reverse transcribed to cDNA by PrimeScript™ RT reagent Kit with gDNA Eraser (Takara, Japan). qRT-PCR was performed utilizing TB Green^®^ Premix Ex Taq™ II (Takara, Japan). Expression of the target genes was computed utilizing the 2^−ΔΔCt^ method with β-actin as the reference gene. Table 1 shows the primers used in qRT-PCR.

**Table 1:** qRT-PCR primers

Gene	Sequence	
STK11	Forward primer	5′-TGTCGGTGGGTATGGACAC-3′
	Reverse primer	5′-CCTTGCCGTAAGAGCCTTCC-3′
β-Actin	Forward primer	5′-CATGTACGTTGCTATCCAGGC-3′
	Reverse primer	5′-CTCCTTAATGTCACGCACGAT-3′

### 2.6 Cell counting kit-8 (CCK-8)

LUAD cells were put at a density of 1 × 10^4^ cells per well in a 96-well plate and cultured under humidified conditions (37℃, 5% CO_2_). The medium was refreshed every 2 days. After 0, 24, 48, and 72 h of incubation, 10 µl of CCK-8 solution was introduced to each well and then followed by incubation at 37℃ for 2 h. The measurement of relative cell viability was performed at 450 nm utilizing a microplate reader.

### 2.7 Flow cytometry

The assay was for ascertaining cell apoptosis. Cells treated with specific conditions for 24 h were gathered and washed with phosphate-buffered saline (PBS). Annexin V-FITC Apoptosis Detection Kit (Beyotime, China) was then used to stain the cells with FITC-conjugated Annexin V and propidium iodide. Apoptotic cells were assayed utilizing a BD FACSCanto II flow cytometer (BD Biosciences, USA). The experiment was fulfilled in triplicate.

### 2.8 Western blot (WB) for protein expression

Cells were collected, washed, and lysed in radioimmunoprecipitation assay buffer (Beyotime, China) containing a protease inhibitor cocktail. Cell lysates (20 μg/lane) were isolated by sodium dodecyl sulfate-polyacrylamide gel electrophoresis and transferred onto polyvinylidene fluoride membranes. Membranes were then kept in 5% skim milk for 1 h and incubated overnight at 4℃ with primary antibodies. Following three washes, the membranes were subjected to incubation with a secondary antibody at room temperature for a duration of 2 h. Visualization was performed using the ECL Plus Ultra-sensitive Chemiluminescence Substrate (Solarbio, China) and a fluorescence and chemiluminescence imaging system (Clinx, China). The primary antibodies used were anti-β-actin (Abcam, UK), anti-GPX4 (Thermo Fisher, USA), anti-SLC7A11 (Thermo Fisher), anti-SCD1 (Abcam), and anti-STK11 (Abcam). The secondary antibody used was goat anti-rabbit IgG H&L (HRP) (Abcam).

### 2.9 Measurement of malondialdehyde (MDA) and glutathione (GSH)

Levels of MDA and GSH in cells were assayed utilizing lipid oxidation assay kit and total GSH assay kit (Beyotime, China), respectively. Absorbance at wavelengths of 532 and 412 nm was quantified utilizing a microplate reader.

### 2.10 Measurement of reactive oxygen species (ROS)

Cells were treated by utilizing ROS Detection Kit (Beyotime, China). Subsequently, cells were kept with DCFH-DA (10 μmol/L) at 37℃ for 20 min. Lipid ROS-positive cells were evaluated using a BD FACSCanto II flow cytometer (BD Biosciences, USA).

### 2.11 Measurement of Fe^2+^ content

Cells were plated at an initial density of 2 × 10^5^ cells per well and kept at 24℃ with 37% CO_2_ for 5 h. Fe^2+^ content was determined using the Fe^2+^ Content Detection Kit (Solarbio) and the absorbance was measured at 593 nm.

### 2.12 Oil red O staining

The staining was performed to assay the lipid droplet (LD) content in LUAD cells. Transfected LUAD cells were kept in a 6-well plate at a density of 5 × 10^4^ cells/mL. After treating with or without CAY10566 (300 nM) for 48 h, cells were rinsed twice with PBS and fixed with 4% paraformaldehyde in the dark for 30 min. Following that, the treated cells were subjected to staining with oil red O solution for a duration of 1 h. The presence of LDs was subsequently visualized and examined utilizing an inverted optical microscope (Olympus, Japan). Lastly, cells were kept with a 100% isopropanol solution to dissolve the lipid-bound red dye, and the absorbance was quantified at a wavelength of 520 nm.

### 2.13 Measurement of MUFAs

Transfected LUAD cells were cultured, and the cell suspension was centrifuged to collect the supernatant. The level of MUFAs was assayed using the Human MUFA Enzyme-linked Immunosorbent Assay Kit (COIBO BIO, China), and the absorbance was measured at 450 nm.

### 2.14 Statistical analysis

Data were expressed as mean ± SD. Multiple group comparisons were conducted using one-way analysis of variance. Comparisons between the two groups were conducted using a *t*-test. All analyses were executed using GraphPad 8.0 software. Statistical significance was defined as a *p*-value of less than 0.05.


**Ethics approval:** No animal/human cell was used.

## 3 Results

### 3.1 STK11 mutation causes aberrant gene expression and increased expression of ferroptosis inhibition-related proteins

**Figure 1:**
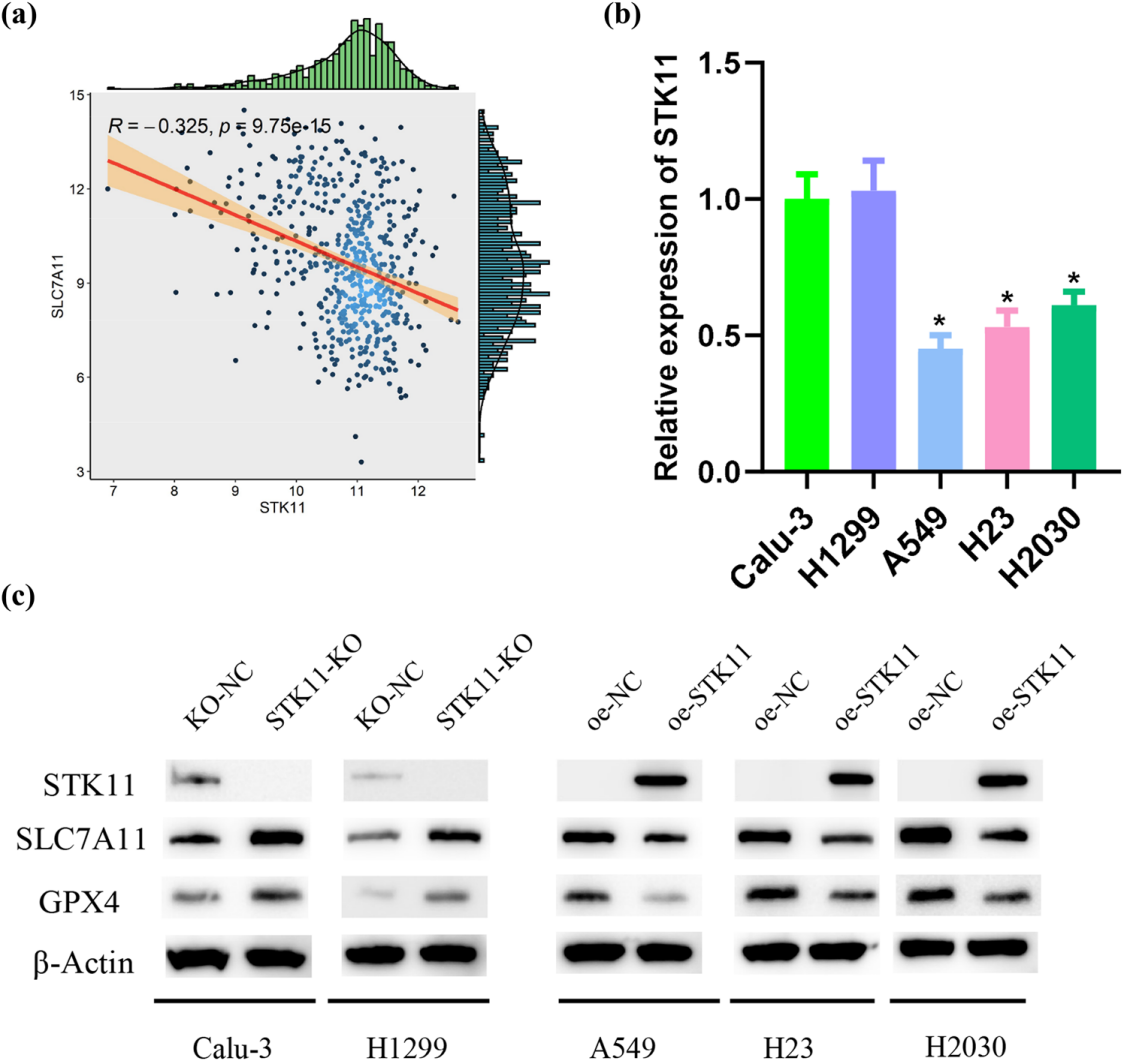
STK11 mutation causes aberrant gene expression and increased expression of ferroptosis inhibition-related proteins. (a) Correlation between STK11 and key ferroptosis inhibition genes; (b) qRT-PCR analysis of STK11 expression in different cell lines; and (c) WB analysis of STK11, SLC7A11, and GPX4 expression levels in cells; * indicates *P* < 0.05.

In our previous study, bioinformatic analysis unmasked a significant negative linkage between STK11 and the key ferroptosis inhibition gene SLC7A11 expression (Figure 1a). STK11 has been found to primarily act as a tumor suppressor in cancer, and loss-of-function mutation in STK11 contributes to cancer progression [19]. Therefore, we tested the expression of STK11 in wild-type LUAD cell lines (Calu-3, H1299, H1975) and STK11-mutant LUAD cell lines (A549, H23, H2030), which displayed a striking downregulation of STK11 expression in STK11-mutant LUAD cell lines (Figure 1b). Subsequently, we employed CRISPR Cas9 technology to construct STK11 knockout vectors (STK11-KO) and empty vectors (NC-KO), which were transfected into STK11 wild-type Calu-3 and H1299 cells, respectively. Additionally, STK11 overexpression vectors were transfected into STK11-mutant LUAD A549, H23, and H2030 cells. WB ascertained a substantial increase in the expression of ferroptosis inhibition-related proteins in LUAD cells after STK11 knockout, while the expression of these proteins was tellingly reduced upon STK11 overexpression (Figure 1c). Findings indicated that STK11 mutation leads to aberrant gene expression and promoted the expression of ferroptosis inhibition-related proteins.

### 3.2 STK11 mutation suppresses ferroptosis in LUAD

**Figure 2:**
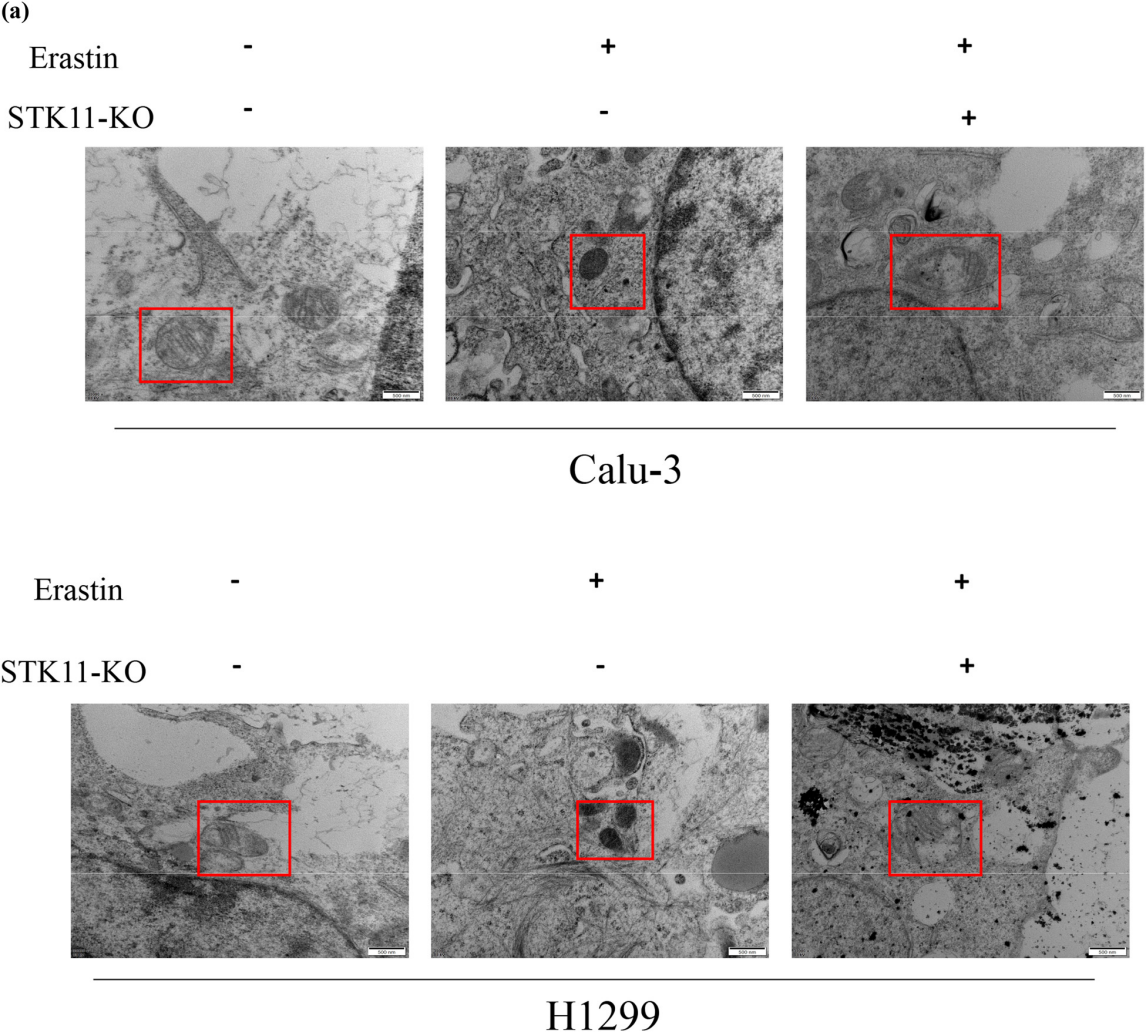

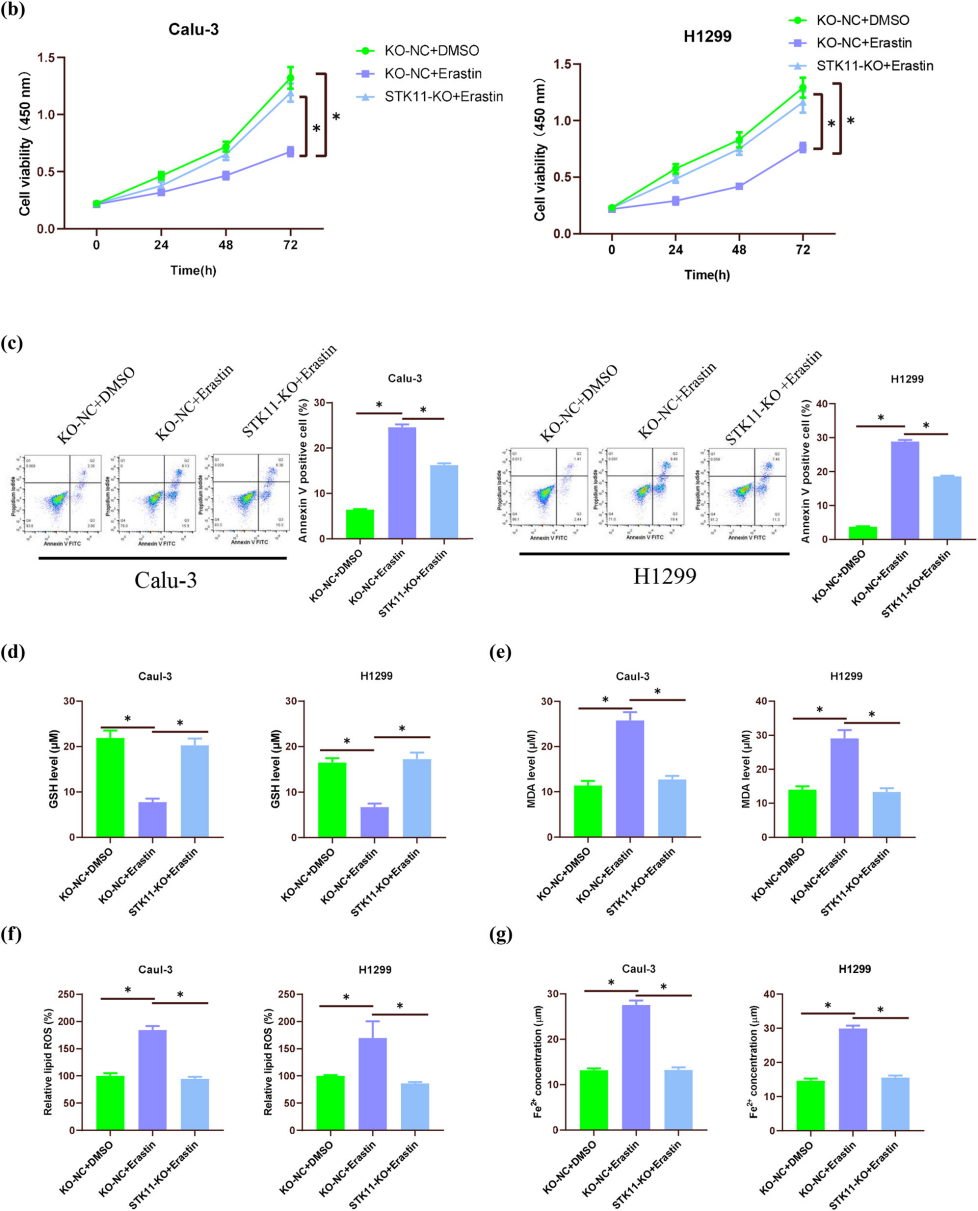
STK11 mutation suppresses ferroptosis in LUAD. (a) TEM observation of mitochondrial morphological changes; (b) CCK-8 analysis of cell viability; (c) flow cytometry analysis of cell apoptosis; (d) GSH assay kit analysis of GSH levels in cells; (e) MDA assay kit analysis of lipid peroxidation levels; (f) ROS assay kit analysis of ROS levels in cells; and (g) Fe^2+^ assay kit analysis of Fe^2+^ levels in cells; * indicates *P* < 0.05.

To further mine how STK11 mutates in the ferroptosis pathway of LUAD cells, we treated KO-NC and STK11-KO transfected cells with Erastin (an inducer of ferroptosis) to induce ferroptosis. TEM observation of mitochondrial morphology revealed that Erastin treatment led to mitochondrial fragmentation, increased membrane density, and decreased cristae in Calu-3 and H1299 cells, while no significant changes in mitochondrial morphology were observed in STK11 knockout cells (Figure 2a). As CCK-8 assay displayed, Erastin treatment notably inhibited the viability of LUAD cells, and STK11 knockout attenuated the inhibitory effect of Erastin on cell viability (Figure 2b). Flow cytometry analysis of apoptosis showed that Erastin treatment increased the number of apoptotic cells in LUAD cells, while STK11 knockout attenuated the pro-apoptotic effect of Erastin (Figure 2c). GSH depletion induces ferroptosis [20], and we detected the GSH levels in cells, which showed that Erastin treatment tellingly reduced GSH levels in cells, while STK11 knockout restored GSH levels in LUAD cells (Figure 2d). MDA content, ROS, and Fe^2+^ levels demonstrated that Erastin treatment significantly increased MDA, ROS, and Fe^2+^ levels in LUAD cells, while STK11 knockout restored the elevated levels induced by Erastin (Figure 2e–g). The obtained results provided evidence suggesting that the presence of the STK11 mutation exerted a suppressive effect on ferroptosis in LUAD.

### 3.3 STK11 mutation affects MUFA synthesis to suppress cell ferroptosis

**Figure 3:**
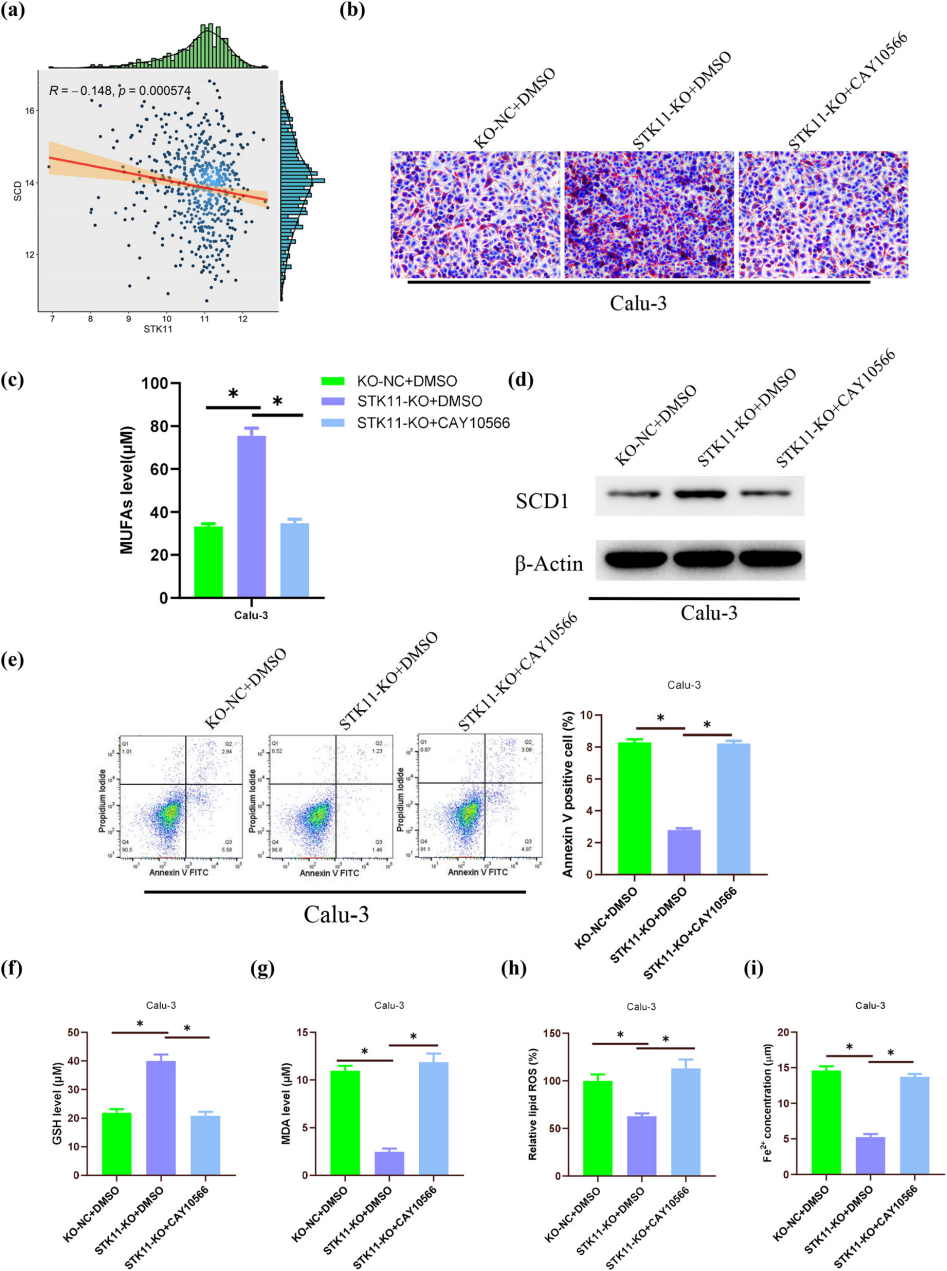
STK11 mutation affects MUFA synthesis to suppress cell ferroptosis. (a) Correlation between STK11 and SCD1; (b) oil red O staining to measure LD content; (c) MUFAs assay kit to quantify MUFA levels; (d) WB analysis of MUFA synthesis-related enzyme protein expression; (e) Flow cytometry analysis of cell apoptosis; (f) GSH assay kit to measure GSH levels in cells; (g) MDA assay kit to measure MDA levels; (h) ROS assay kit to measure ROS levels in cells; and (i) Fe^2+^ assay kit to measure Fe^2+^ levels in cells; * indicates *P* < 0.05.

Further bioinformatic analysis revealed a significant negative correlation between STK11 and SCD (Figure 3a). SCD (also known as SCD1) is a key rate-limiting enzyme in lipid metabolism that catalyzes the synthesis of MUFAs by introducing double bonds into acyl-CoA chains [21]. To investigate the impact of STK11 mutation on the MUFA synthesis pathway, we treated LUAD cells with the SCD1 inhibitor CAY10566. Oil red O staining for LD distribution exhibited a substantial increase in LD content in LUAD cells after STK11 knockout, which was attenuated by the CAY10566 treatment (Figure 3b). Lipids were extracted from cancer cells utilizing a lipid extraction kit, and MUFA content (unsaturated FAs) was quantified using a lipid quantification method. The outcomes demonstrated a significant elevation in MUFA levels in LUAD cells after STK11 knockout, which was suppressed by CAY10566 treatment (Figure 3c). WB analysis of the expression of the MUFA synthesis-related enzyme SCD1 showed that STK11 knockout significantly promoted SCD1 protein expression in LUAD cells, and CAY10566 attenuated the stimulatory effect of STK11 knockout on SCD1 expression (Figure 3d). Flow cytometry analysis investigating the influence of MUFA synthesis on cell apoptosis demonstrated a noteworthy decrease in the population of apoptotic cells in LUAD cells following STK11 knockout, while CAY10566 attenuated the inhibitory effect of STK11 knockout on cell apoptosis (Figure 3e). Subsequently, we measured the GSH levels in cells and found a significant increase in GSH levels after STK11 knockout, which could be restored by CAY10566 in LUAD cells (Figure 3f). MDA content, ROS, and Fe^2+^ levels showed that STK11 knockout significantly inhibited MDA, ROS, and Fe^2+^ levels in LUAD cells, while CAY10566 restored the decreased levels induced by STK11 knockout (Figure 3g–i). These results indicated that STK11 mutation affects MUFA synthesis, thereby suppressing cell ferroptosis.

## 4 Discussion

LUAD stands as a primary contributor to cancer-related deaths, and its incidence is progressively rising [22]. Currently, it is well-established that lipid peroxidation-induced ferroptosis plays a pivotal role in tumor progression [23,24,25]. In this study, we revealed that STK11 mutations negatively regulated ferroptosis in LUAD cells by regulating MUFA synthesis. STK11 mutations promoted the expression of ferroptosis inhibition-related proteins in LUAD cells and inhibited cellular ferroptosis. Further in-depth analysis revealed that STK11 mutations promoted MUFA synthesis, leading to the down-regulation of ferroptosis in LUAD cells. These findings underscored the potential of targeting the STK11 mutation as a therapeutic strategy to modulate the ferroptosis pathway for the treatment of LUAD.

Despite ongoing improvements in treatment approaches, the survival rate for LUAD patients remains low [26]. Tumor size plays a pivotal role in determining the staging and treatment options, exerting a substantial impact on patient prognosis [27]. The imbalance between tumor growth and cell death represents a pivotal factor in the progression of tumors. Recent studies have highlighted that the induction of ferroptosis in tumor cells can effectively impede tumor growth [28,29]. For example, in human glioma, amentoflavone inhibits tumor cell proliferation and triggers cell death by promoting autophagy-dependent iron-dependent cell death [30]. In hepatocellular carcinoma (HCC), polyphyllin VI hinders STAT3 phosphorylation, suppresses GPX4 expression and triggers ferroptosis in HCC cells, ultimately hindering invasion and metastasis [31]. Here, we ascertained a negative linkage between STK11 and the key inhibitor of ferroptosis gene SLC7A11, suggesting that STK11 mutation suppresses ferroptosis in LUAD cells. STK11 (LKB1) is a tumor suppressor gene that is commonly lost or inactivated in LC patients. An illustrative example is the CheckMate-4 phase III trial, where STK11/LKB1 mutations are identified as the prevailing genomic driver responsible for primary resistance to PD-1 axis inhibitors in KRAS-mutant LUAD [16]. Therefore, it is considered a pivotal gene in the progression of LC [32,33]. Nevertheless, the precise molecular mechanisms through which STK11 mutations influence ferroptosis in LUAD cells have yet to be fully elucidated.

To delve into the molecular mechanisms underlying the impact of STK11 mutation on ferroptosis in LUAD, we conducted further bioinformatics analysis and discovered a negative correlation between SCD1 and STK11. SCD1 serves as a crucial regulator in the pathway of FA metabolism, converting saturated FAs into MUFAs and suppressing ferroptosis [34]. In esophageal squamous cell carcinoma, BACH1 negatively regulates the expression of SCD1 to inhibit MUFA generation, thereby inducing ferroptosis and facilitating lymph node metastasis [35]. In HCC, hydroxycarboxylic acid receptor 1/monocarboxylate transporter 1 activation through lactate uptake blockade activates AMPK, leading to the downregulation of SCD1 and further induction of ferroptosis [36]. Additionally, Wohlhieter et al. [18] revealed significantly elevated SCD1 expression in cells with co-occurring STK11 and KEAP1 mutations, enhancing LC cells’ resistance to ferroptosis and promoting cell proliferation. These studies collectively demonstrate the influence of lipid metabolism on ferroptosis. In our research, we ascertained that STK11 mutation fostered SCD1 expression and MUFA synthesis, thereby inhibiting ferroptosis. Our findings provided a novel and effective target for promoting ferroptosis in cancer cells by targeting MUFA synthesis, offering new strategies in treating LUAD.

Moreover, our work first indicated that STK11 mutation inhibited ferroptosis in LUAD cells through the promotion of MUFA synthesis. Furthermore, through a review of the literature, we identified that the level of GSH was also an important factor influencing ferroptosis, although its molecular mechanisms remain incompletely understood. Therefore, further exploration in this area is warranted. In summary, our research suggested that SLC7A11 and SCD1 could potentially be effective in treating LUAD with STK11 mutation, and we hoped that our findings can assist the advancement of novel therapeutic strategies for LUAD.

## References

[1] Sung H, Ferlay J, Siegel RL, Laversanne M, Soerjomataram I, Jemal A, et al. Global cancer statistics 2020: GLOBOCAN estimates of incidence and mortality worldwide for 36 cancers in 185 countries. CA Cancer J Clin. 2021;71(3):209–49. 10.3322/caac.21660 33538338

[2] Ettinger DS, Wood DE, Aisner DL, Akerley W, Bauman JR, Bharat A, et al. NCCN guidelines insights: Non-small cell lung cancer, version 2.2021. J Natl Compr Canc Netw. 2021;19(3):254–66. 10.6004/jnccn.2021.0013 33668021

[3] Duma N, Santana-Davila R, Molina JR. Non-small cell lung cancer: Epidemiology, screening, diagnosis, and treatment. Mayo Clin Proc. 2019;94(8):1623–40. 10.1016/j.mayocp.2019.01.013 31378236

[4] Tang D, Chen X, Kang R, Kroemer G. Ferroptosis: Molecular mechanisms and health implications. Cell Res. 2021;31(2):107–25. 10.1038/s41422-020-00441-1 PMC8026611 33268902

[5] Jiang X, Stockwell BR, Conrad M. Ferroptosis: Mechanisms, biology and role in disease. Nat Rev Mol Cell Biol. 2021;22(4):266–82. 10.1038/s41580-020-00324-8 PMC8142022 33495651

[6] Ouyang S, Li H, Lou L, Huang Q, Zhang Z, Mo J, et al. Inhibition of STAT3-ferroptosis negative regulatory axis suppresses tumor growth and alleviates chemoresistance in gastric cancer. Redox Biol. 2022;52:102317. 10.1016/j.redox.2022.102317 PMC9108091 35483272

[7] Chen H, Wen J. Iron oxide nanoparticles loaded with paclitaxel inhibits glioblastoma by enhancing autophagy-dependent ferroptosis pathway. Eur J Pharmacol. 2022;921:174860. 10.1016/j.ejphar.2022.174860 35278406

[8] Bi G, Liang J, Zhao M, Zhang H, Jin X, Lu T, et al. miR-6077 promotes cisplatin/pemetrexed resistance in lung adenocarcinoma via CDKN1A/cell cycle arrest and KEAP1/ferroptosis pathways. Mol Ther Nucleic Acids. 2022;28:366–86. 10.1016/j.omtn.2022.03.020 PMC9035384 35505963

[9] Dierge E, Debock E, Guilbaud C, Corbet C, Mignolet E, Mignard L, et al. Peroxidation of n-3 and n-6 polyunsaturated fatty acids in the acidic tumor environment leads to ferroptosis-mediated anticancer effects. Cell Metab. 2021;33(8):1701–15e5. 10.1016/j.cmet.2021.05.016 34118189

[10] Dixon SJ, Stockwell BR. The hallmarks of ferroptosis. 2019;3(1):35–54. 10.1146/annurev-cancerbio-030518-055844 PMC12851585 41613499

[11] Sun LL, Linghu DL, Hung MC. Ferroptosis: A promising target for cancer immunotherapy. Am J Cancer Res. 2021;11(12):5856–63. PMC8727800 35018229

[12] Mortensen MS, Ruiz J, Watts JL. Polyunsaturated fatty acids drive lipid peroxidation during ferroptosis. Cells. 2023;12(5):804. 10.3390/cells12050804 PMC10001165 36899940

[13] Magtanong L, Ko PJ, To M, Cao JY, Forcina GC, Tarangelo A, et al. Exogenous monounsaturated fatty acids promote a ferroptosis-resistant cell state. Cell Chem Biol. 2019;26(3):420–32 e9. 10.1016/j.chembiol.2018.11.016 PMC6430697 30686757

[14] Gleeson FC, Kipp BR, Levy MJ, Voss JS, Campion MB, Minot DM, et al. Somatic STK11 and concomitant STK11/KRAS mutational frequency in stage IV lung adenocarcinoma adrenal metastases. J Thorac Oncol. 2015;10(3):531–4. 10.1097/JTO.0000000000000391 25695224

[15] Della Corte CM, Byers LA. Evading the STING: LKB1 loss leads to STING silencing and immune escape in KRAS-mutant lung cancers. Cancer Discov. 2019;9(1):16–8. 10.1158/2159-8290.CD-18-1286 PMC8330553 30626603

[16] Skoulidis F, Goldberg ME, Greenawalt DM, Hellmann MD, Awad MM, Gainor JF, et al. STK11/LKB1 mutations and PD-1 inhibitor resistance in KRAS-mutant lung Adenocarcinoma. Cancer Discov. 2018;8(7):822–35. 10.1158/2159-8290.CD-18-0099 PMC6030433 29773717

[17] Koyama S, Akbay EA, Li YY, Aref AR, Skoulidis F, Herter-Sprie GS, et al. STK11/LKB1 deficiency promotes neutrophil recruitment and proinflammatory cytokine production to suppress T-cell activity in the lung tumor microenvironment. Cancer Res. 2016;76(5):999–1008. 10.1158/0008-5472.CAN-15-1439 PMC4775354 26833127

[18] Wohlhieter CA, Richards AL, Uddin F, Hulton CH, Quintanal-Villalonga A, Martin A, et al. Concurrent mutations in STK11 and KEAP1 promote ferroptosis protection and SCD1 dependence in lung cancer. Cell Rep. 2020;33(9):108444. 10.1016/j.celrep.2020.108444 PMC7722473 33264619

[19] Pons-Tostivint E, Lugat A, Fontenau JF, Denis MG, Bennouna J. STK11/LKB1 modulation of the immune response in lung cancer: From biology to therapeutic impact. Cells. 2021;10(11):3129. 10.3390/cells10113129 PMC8618117 34831355

[20] Sun Y, Zheng Y, Wang C, Liu Y. Glutathione depletion induces ferroptosis, autophagy, and premature cell senescence in retinal pigment epithelial cells. Cell Death Dis. 2018;9(7):753. 10.1038/s41419-018-0794-4 PMC6037763 29988039

[21] Ascenzi F, De Vitis C, Maugeri-Sacca M, Napoli C, Ciliberto G, Mancini R. SCD1, autophagy and cancer: Implications for therapy. J Exp Clin Cancer Res. 2021;40(1):265. 10.1186/s13046-021-02067-6 PMC8383407 34429143

[22] Siegel RL, Miller KD, Jemal A. Cancer statistics, 2020. CA Cancer J Clin. 2020;70(1):7–30. 10.3322/caac.21590 31912902

[23] Gong C, Ji Q, Wu M, Tu Z, Lei K, Luo M, et al. Ferroptosis in tumor immunity and therapy. J Cell Mol Med. 2022;26(22):5565–79. 10.1111/jcmm.17529 PMC9667519 36317423

[24] Zhang Y, Zhuang L, Gan B. BAP1 suppresses tumor development by inducing ferroptosis upon SLC7A11 repression. Mol Cell Oncol. 2019;6(1):1536845. 10.1080/23723556.2018.1536845 PMC6370386 30788415

[25] Lei G, Zhang Y, Koppula P, Liu X, Zhang J, Lin SH, et al. The role of ferroptosis in ionizing radiation-induced cell death and tumor suppression. Cell Res. 2020;30(2):146–62. 10.1038/s41422-019-0263-3 PMC7015061 31949285

[26] Strano S, Lupo A, Lococo F, Schussler O, Loi M, Younes M, et al. Prognostic significance of vascular and lymphatic emboli in resected pulmonary adenocarcinoma. Ann Thorac Surg. 2013;95(4):1204–10. 10.1016/j.athoracsur.2012.12.024 23415237

[27] Travis WD, Asamura H, Bankier AA, Beasley MB, Detterbeck F, Flieder DB, et al. The IASLC lung cancer staging project: Proposals for coding T categories for Subsolid nodules and assessment of tumor size in part-solid tumors in the forthcoming eighth edition of the TNM Classification of lung cancer. J Thorac Oncol. 2016;11(8):1204–23. 10.1016/j.jtho.2016.03.025 27107787

[28] Su Y, Zhao B, Zhou L, Zhang Z, Shen Y, Lv H, et al. Ferroptosis, a novel pharmacological mechanism of anti-cancer drugs. Cancer Lett. 2020;483:127–36. 10.1016/j.canlet.2020.02.015 32067993

[29] Ye Z, Zhuo Q, Hu Q, Xu X, Mengqi L, Zhang Z, et al. FBW7-NRA41-SCD1 axis synchronously regulates apoptosis and ferroptosis in pancreatic cancer cells. Redox Biol. 2021;38:101807. 10.1016/j.redox.2020.101807 PMC7710650 33271455

[30] Chen Y, Li N, Wang H, Wang N, Peng H, Wang J, et al. Amentoflavone suppresses cell proliferation and induces cell death through triggering autophagy-dependent ferroptosis in human glioma. Life Sci. 2020;247:117425. 10.1016/j.lfs.2020.117425 32057904

[31] Huang Q, Li J, Ma M, Lv M, Hu R, Sun J, et al. High‑throughput screening identification of a small‑molecule compound that induces ferroptosis and attenuates the invasion and migration of hepatocellular carcinoma cells by targeting the STAT3/GPX4 axis. Int J Oncol. 2023;62(3):42. 10.3892/ijo.2023.5490 PMC9946807 36825585

[32] Park C, Lee Y, Je S, Chang S, Kim N, Jeong E, et al. Overexpression and selective anticancer efficacy of ENO3 in STK11 mutant lung cancers. Mol Cell. 2019;42(11):804–9. 10.14348/molcells.2019.0099 PMC6883975 31697874

[33] Facchinetti F, Bluthgen MV, Tergemina-Clain G, Faivre L, Pignon JP, Planchard D, et al. LKB1/STK11 mutations in non-small cell lung cancer patients: Descriptive analysis and prognostic value. Lung Cancer. 2017;112:62–8. 10.1016/j.lungcan.2017.08.002 29191602

[34] Sen U, Coleman C, Sen T. Stearoyl coenzyme A desaturase-1: Multitasker in cancer, metabolism, and ferroptosis. Trends Cancer. 2023;9(6):480–9. 10.1016/j.trecan.2023.03.003 37029018

[35] Xie X, Tian L, Zhao Y, Liu F, Dai S, Gu X, et al. BACH1-induced ferroptosis drives lymphatic metastasis by repressing the biosynthesis of monounsaturated fatty acids. Cell Death Dis. 2023;14(1):48. 10.1038/s41419-023-05571-z PMC9860034 36670112

[36] Zhao Y, Li M, Yao X, Fei Y, Lin Z, Li Z, et al. HCAR1/MCT1 regulates tumor ferroptosis through the lactate-mediated AMPK-SCD1 activity and its therapeutic implications. Cell Rep. 2020;33(10):108487. 10.1016/j.celrep.2020.108487 33296645

